# Comment on the Nanoparticle Conclusions in Crüts *et al*. (2008), "Exposure to diesel exhaust induces changes in EEG in human volunteers"

**DOI:** 10.1186/1743-8977-5-10

**Published:** 2008-07-24

**Authors:** Peter A Valberg, Christopher M Long, Thomas W Hesterberg

**Affiliations:** 1 Gradient Corporation, 20 University Road, Cambridge, MA, USA; 2 Navistar Incorporated, Warrenville, IL, USA

## Abstract

A recent publication in this journal reported interesting changes in electroencephalographic (EEG) waves that occurred in 10 young, male volunteers following inhalation for one hour of elevated levels of diesel-engine exhaust fumes [1]. The authors then proposed a chain of causal events that they hypothesized underlay their observed EEG changes. Their reasoning linked the observed results to nanoparticles in diesel-engine exhaust (DEE), and went on to suggest that associations between changes in ambient particulate matter (PM) levels and changes in health statistics might be due to the effects of diesel-engine exhaust (DEE) nanoparticles on EEG. We suggest that the extrapolations of the Crüts *et al*. EEG findings to casual mechanisms about how ambient levels of DEE particulate might affect electrical signals in the brain, and subsequently to how DEE particulate might alter disease risk, are premature.

## Commentary

In their recent publication, "Exposure to Diesel Exhaust Induces Changes in EEG in Human Volunteers," Crüts *et al*. [1] exposed 10 healthy, young male volunteers to whole diesel-engine exhaust (DEE) (at DEE particulate levels of 300 μg/m^3 ^and at gaseous DEE constituents levels of 1.6 ppm NO_2_, 4.5 ppm NO, 7.5 ppm CO, and 4.3 ppm total hydrocarbons). During exposure, and for one hour following, brain electrical activity was monitored using quantitative electroencephalography at 8 different sites on the scalp, and the EEG frequency spectrum was monitored (Hz = Hertz = cycles per second, a measure of the frequency). The EEG contains electrical brain signals of many different frequencies ranging perhaps from about 1 Hz to 50 Hz, and the median power frequency (MPF) is that frequency, over the whole spectrum of frequencies, where half the EEG signal power is below this frequency and half the EEG signal power is above this frequency.

The authors reported that, using 3-minute-duration measurements, and after averaging over the 10 subjects, the median power frequency of the EEG measured at a frontal head location increased from 8.5 Hz to 9.0 Hz during sham exposure, and increased from 8.2 Hz to 10.1 Hz following DEE exposure. At a more central head location, the average EEG median frequency was stable during sham exposure (9.9 Hz to 9.8 Hz), but during DEE exposure the median frequency increased (8.2 Hz to 9.7 Hz). The SD of these group averages ranged from ± 1.1 to ± 2.7 Hz, and although the group-average frequency shifts measured following DEE exposure reached statistical significance, not every subject showed this pattern, and in fact several showed a reverse pattern (*i.e.*, greater frequency shift during sham exposure than during DEE exposure).

Although the authors acknowledge that these subtle changes in EEG frequencies may be mediated by a variety of different pathways (including a variety of chemicals stimulating olfactory receptors), they conclude that "*our findings are due to an effect of nanoparticles that slowly penetrate the brain or affect neurophysiologic signaling*." That is, they propose the following series of explanatory events: (1) DEE includes particles having diameters in the range of 1 to 100 nanometers (10^-9 ^m), (2) these DEE nanoparticles do not aggregate into larger particles prior to inhalation, (3) the nanoparticles deposit on the nasal epithelium of the volunteers, (4) the nanoparticles are taken up into olfactory sensory cells, (5) the nanoparticles translocate to the brain *via *the olfactory nerve, (6) in the brain, the nanoparticles contribute to oxidative stress, (7) the brain is very sensitive to oxidative stress, and this is demonstrated by the reported changes in EEG, and (8) these changes may be a harbinger of more serious diseases such as Parkinson's and Alzheimer's and cognitive function decrease.

We propose that extrapolation of the reported measurements of brain electrical activity (using whole DEE with significant amounts of CO_2_, NO_x_, CO, hydrocarbons, aldehydes, *etc.*) to the potential role of DEE particulate in reported epidemiologic associations between ambient PM levels and health-effect outcomes is not warranted at our present state of knowledge. Recognizing that the Crüts *et al*. findings may be relied upon to project the health risks of environmental DEE exposures, we feel it necessary to call attention to several key study limitations and uncertainties that affect the broader health implications of these study findings.

Although the study authors acknowledge several limitations and uncertainties of their study and the conclusions they draw from it, some points deserve additional note. The diesel engine (idling Volvo TD45, model year 1991, 4.5-liter, 4 cylinder, 680 rpm, diesel engine) that served as the source of the DEE exposures is nearly 20 years old – a very old technology by today's standards, and thus the DEE produced cannot be expected to represent the present-day fleet of cleaner-burning diesel engines. The specific diesel fuel used was not specified, but this factor also modifies DEE. Due to progressive improvements in diesel-engine technology over the last two decades, today's New Technology Diesel Exhaust (NTDE) emissions contain minimal levels of PM and are physically and chemically very different from DEE from earlier diesel technologies [2,3]. To better understand the relevance of the Crüts *et al*. study findings for health assessments of current and future DEE, one would need to see how the results of their experiments would turn out for NTDE emissions.

Furthermore, it would have been helpful if the study authors compared the elevated nature of the volunteer exposures to more typical DEE exposure levels that an individual would commonly encounter in his/her everyday life. For example, with the exception of coal-mining, where elemental carbon concentrations as high as 200 to 400 μg/m^3 ^have been measured [2], occupational monitoring indicates that a DEE particulate concentration of 300 μg/m^3 ^exceeds typical DEE particulate levels that a worker might encounter, including railroad-worker populations, public transit workers and airport crews, mechanics and dock workers, and long- and short-haul truck drivers [2,4]. In addition, the Crüts *et al*. exposure levels exceed by far the pollutant levels recently reported by McCreanor *et al*. [5] for street-level measurements made along heavily-trafficked London streets, with the comparisons for three parameters shown in Table 1.

**Table 1:** Comparison of ambient levels of particles (PM) and NO_x _to levels used by Crüts *et al*.

	**McCreanor *et al*. **[5]	**Crüts *et al*. **[1]
**PM**_2.5_	28 μg/m^3^	300 μg/m^3^
**Particle #**	640,000 particles/cm^3^	1,200,000 particles/cm^3^
**NO**_2_	80 ppb	1,600 ppb

Given the highly elevated nature of the exposure levels of both DEE particulate and gaseous DEE constituents in the Crüts *et al*. study, it is highly speculative at this time to draw conclusions regarding the specific agent underlying the observed responses, such as DEE nanoparticles. In fact, the authors do not report what fraction of the 300 μg/m^3 ^DEE particulate mass falls in the "nanoparticle" size range.

While the authors acknowledge many difficulties in linking short-term brain electrical activity changes with clinically relevant health effects, they could have provided additional perspective for weighing the potential health significance of their findings. In particular, they might have discussed how similar, and even greater, changes in brain activity are common in our everyday lives. For example, exercise [6]; intake of caffeine, alcohol, refined sugar, and nicotine; and changes in cognitive demands, such as attention and concentration, mental loads and stress, and sleep/sleep deprivation all modify EEG [7]. In fact, variability in brain activity is normal in the absence of defined stimulation, as demonstrated by Cummings *et al*. [7] who provided evidence of the diurnal variation in the cortical quantitative EEG in a study of 18 healthy volunteers. To better understand the potential health implications of the Crüts *et al*. findings, it is important to consider not only how the observed reversible changes in brain activity compare to those associated with other common stimuli, but also to normal physiological ranges encountered in everyday life.

In conclusion, the novel findings of Crüts *et al*. merit follow-up studies. To elucidate a precise mechanism, as suggested by the study authors, one needs to determine whether the observed responses are mediated by sensory elements, stress responses, gaseous DEE constituents (some of which, *e.g.*, NO, are neurologically active), DEP nanoparticles, or other factors. With regard to the potential effects of inhaled nanoparticles, we agree with the study authors that more work is needed to determine whether other types of nanoparticles (*e.g.*, viruses, cooking fumes, engineered nanoparticles) elicit similar responses on brain activity, given that nano-sized DEE particles would not be unique in the potential for central nervous system translocation. We would add that follow-up studies should also be conducted at lower levels of NTDE and at multiple exposure levels to investigate dose-response. Until these types of studies have been conducted and there is confirmatory evidence that these EEG changes are markers for "oxidative stress" in the brain or cognition function (*e.g.*, measured *via *cognitive performance tests, magnetic resonance imaging), we feel the data thus far do not support designating the reported effects on brain electrical activity as a known adverse health effect, or established health hazard, of environmental DEE exposure.

In summary, readers of "*Particle and Fibre Toxicology*" should recognize that the physiological and neurological effects observed following inhalation at elevated levels of a complex mixture of chemicals and particles such as DEE can be many, and the potential mechanistic pathways by which these effects could arise are likewise manifold. Consequently, concluding that EEG alterations *per se *are likely due to the nanoparticle content in DEE, although provocative, seems premature.

## Competing interests

PAV and CML work at Gradient Corporation, which provides technical consulting to government, regulators, and regulated industries. TWH is employed by Navistar, Incorporated.

## Authors' contributions

PAV, CML, TWH all contributed to writing this commentary "Letter to the Editor" regarding the 2008 Crüts *et al*. article in *PF&T *[1]. All authors read and approved the final manuscript.
